# Visual Tracking Based on Extreme Learning Machine and Sparse Representation

**DOI:** 10.3390/s151026877

**Published:** 2015-10-22

**Authors:** Baoxian Wang, Linbo Tang, Jinglin Yang, Baojun Zhao, Shuigen Wang

**Affiliations:** 1 School of Information and Electronics, Beijing Institute of Technology, Beijing 100081, China; E-Mails: wbx1025@bit.edu.cn (B.W.); yangjinglin@bit.edu.cn (J.Y.); zbj@bit.edu.cn (B.Z.); sgwang@bit.edu.cn (S.W.); 2 Beijing Key Laboratory of Embedded Real-Time Information Processing Technology, Beijing 100081, China

**Keywords:** visual tracking, extreme learning machine, sparse representation, manifold learning, accelerated proximal gradient

## Abstract

The existing sparse representation-based visual trackers mostly suffer from both being time consuming and having poor robustness problems. To address these issues, a novel tracking method is presented via combining sparse representation and an emerging learning technique, namely extreme learning machine (ELM). Specifically, visual tracking can be divided into two consecutive processes. Firstly, ELM is utilized to find the optimal separate hyperplane between the target observations and background ones. Thus, the trained ELM classification function is able to remove most of the candidate samples related to background contents efficiently, thereby reducing the total computational cost of the following sparse representation. Secondly, to further combine ELM and sparse representation, the resultant confidence values (*i.e*., probabilities to be a target) of samples on the ELM classification function are used to construct a new manifold learning constraint term of the sparse representation framework, which tends to achieve robuster results. Moreover, the accelerated proximal gradient method is used for deriving the optimal solution (in matrix form) of the constrained sparse tracking model. Additionally, the matrix form solution allows the candidate samples to be calculated in parallel, thereby leading to a higher efficiency. Experiments demonstrate the effectiveness of the proposed tracker.

## 1. Introduction

As one of the fundamental topics in computer vision and related fields, visual tracking has been playing a key role in many applications, such as video surveillance, motion recognition, traffic monitoring, and so on. Although many tracking methods have been proposed in recent years [1,2,3,4,5,6,7,8], it is still a challenging problem due to numerous factors, including illumination variations, pose changes, partial occlusions and background clutter, just to name a few.

Recently, sparse representation [9] has been successfully applied to image restoration [10], target detection [8], face recognition [11], and so on. Xue *et al.* [12] firstly introduced sparse representation into visual tracking, in which the tracked objects could be sparsely represented via a linear combination of the target templates (accounting for the object) and the trivial ones (accounting for the background noise). Thus, combined with the particle filter framework, the tracking task is formulated as solving a l1 norm optimization problem (named as L1 tracker) of one under-determined linear system, and the likelihood of particle belonging to the true object is calculated according to its error from being reconstructed in the target templates. For L1 tracker, the number of particles equals the number of the l1 norm minimizations for particles’ sparse representation. Hence, the number of particles dominates the total calculation cost of L1 tracker, and the resulting computational burden hinders its real-time applications. On the other hand, a small number of particles could not account for the dynamic changes of a moving object. Therefore, how to balance the efficiency and robustness is a difficult issue for sparse representation-based trackers.

For the efficiency aspect, in [13], a minimum error bounded particle resampling method is advocated for removing the candidate samples, which have large reconstruction errors in the target templates, thereby reducing the amount of l1 norm minimizations. Bao *et al.* [14] also exploited a fast accelerated approach to boost the tracking efficiency of L1 tracker. However, these adopted strategies only take into account the foregone target observations, and the corresponding discriminative capability is not enough for complex environments. Therefore, some potential candidate samples may be abandoned, which results in a loss of tracking accuracy. Moreover, owing to that the information from backgrounds being ignored, there exist some surplus particle samples similar to the backgrounds, and the tracking speed is still restricted.

From the robustness point of view, several methods have also extended the L1 tracker, such as mining the interdependencies between candidates [15], using orthogonal basis vectors instead of raw pixel templates [16], and so on. These trackers have obtained some improvements in terms of tracking accuracy, but they do not take surrounding visual context into account as well as [13,14] and discard useful information that can be exploited to better separate the target object from the backgrounds.

In order to improve the robustness of sparse representation-based trackers, some scholars introduced the background information into the tracking framework. In [17], the trivial templates are replaced with the background templates, and the object candidate should have the highest comparison between the foreground and backgrounds. However, the appearance changes of the backgrounds are very unstable. Therefore, the sparse constraints upon the background samples may not have a good performance. Motivated by [17], Zhuang *et al.* [18] reversely find the sparse representation of the target and backgrounds in the candidate samples space and achieve some good results. However, numerous candidates are required for ensuring the over-complete property of the candidate sample space, thereby tending to be inefficient. Besides, to further boost the tracking robustness, the manifold learning constraint is introduced in [18,19]. For manifold learning, the correlation matrix between the samples for sparse representation is calculated in advance by the K-nearest neighbor (KNN) technique [20]. However, computing from KNN is also a time-consuming task, which is not suitable for real-time tracking.

Through the above analysis, the existing sparse representation-based trackers often suffer from low efficiency and poor robustness, which can be attributed to the following two aspects: (1) most of the candidate samples participating in the sparse representation are from backgrounds, which dominate the total computational burden of the sparsity model; (2) the useful comparison information between the foreground and its surroundings is not utilized, which causes the tracker to not be robust in complicated backgrounds. To address these issues, we attempt to propose a new coarse-to-fine visual tracking framework, and the incipient motivations are shown in the following Section 1.1.

### 1.1. Motivations

In this subsection, we present the detailed motivations in terms of the computational burden, the robustness aspect, the selection of the classifier and the further combination for the extreme learning machine (ELM) and sparse representation.

From the computational burden point of view, as mentioned above, the majority of the input candidate samples belong to the background, which leads to low tracking speed for the traditional sparsity trackers. In [13], the minimum error bounded strategy has been applied for removing the useless background candidates, which have large reconstruction errors in the target template space. However, as we empirically observed, some potential target samples may also have poor representation in the target template space and then be discarded. On the other hand, the reserved candidate samples still contain the background contents. Thus, only by using the target template space for reducing candidates, the resultant tracking may not be robust and efficient. How to effectively cut down the number of background samples is important for the tracker in practical applications. Recently, there has been much progress in the classifier model, and researchers found that the classifier could perform robustly in distinguishing one object from other ones [21,22]. Therefore, the classification technique can be used to find the optimal separate hyperplane between the target and its backgrounds. Utilizing the differences between the target and its surroundings, the tracker can effectively remove the background samples and select the potential object ones for the following sparse representation. Compared to the minimum error bounded strategy, the classification model would be superior and can be treated as the prior process before the sparse representation.

As for the robustness aspect, the sparsity-based trackers can be effective when dealing with the background noise and illumination changes. However, the traditional sparsity tracking framework only considers the foregone target observations and then does not performs well in some challenging environments (e.g., motion blur, low object-background contrast). In these cases, by using the classification technique, the resulting tracker could find the potential candidate regions and alleviate the disturbances from different background contents. Furthermore, the used classifier can handle the pose variations of object well, which further contributes to robust tracking.

For real-time and robust visual tracking, the adopted classification technique should have a low computational burden and quickly adapt to dynamic visual changes. However, most of the existing classifiers cannot achieve optimal performances in terms of learning accuracy and training speed. For instance, the support vector machine (SVM)-based models [21,22] often have good performances, but suffer from high computational burden owing to the heavy quadratic programming. On the other hand, the naive Bayes-based ones [23,24] have a fast implementation, while not being able to achieve satisfactory performance, because of the weakness of simple classifiers. In this work, we attempt to use an emergent learning technique, *i.e*., ELM. The ELM proposed by Huang *et al.* [25,26] was originally developed for training single hidden layer feed-forward neural networks (SLFNs). Compared to traditional methods (neural networks or SVM), ELM not only has a faster learning speed, but also obtains a better generalization in many applications, such as face recognition [27], object localization [28], data fusion [29], and so on.

Via using the simple cascading combination of ELM and sparse representation, some good tracking results can be obtained. However, we hope to find the further interaction between the above two different models. As mentioned before, the manifold learning constraint (MLC) could boost the sparsity tracking performances [18,19]. Unfortunately, the calculation of the correlation matrix in MLC generally depends on the time-consuming KNN method. From the technique point of view, both ELM and KNN belong to the same machine learning category. Thus, we can also apply the ELM model for computing the correlation matrix in MLC. It is noted that the learning results of samples on the ELM classification can be directly exploited to construct the MLC, which is preferred for real-time visual tracking.

### 1.2. Contributions

Motivated by the above analysis and discussion, we attempt to apply the novel ELM technique into the sparse tracking model and achieve better tracking performances in terms of efficiency and robustness. To our knowledge, this is the first time that the extreme learning machine is combined into the sparsity tracking framework. Our contributions can be summed up in the following three aspects:

(1) To make full use of the comparison information between the foreground and its backgrounds, ELM is used to find the decision boundary between the target observations and background ones. Then, the trained ELM classification function is applied for removing most of the candidate samples similar to the background contents. The ELM method is discriminative enough for distinguishing the target and backgrounds with a fast learning speed [30]. Thus, the few best potential candidate samples related to the object views can be quickly selected for the following sparse representation, which can decrease the total computational cost of the tracking algorithm.

(2) For further developing the combination of ELM and sparse representation, a novel constrained sparse tracking model is built. Specifically, the output values of candidate samples on the ELM classification function are to construct a new manifold learning constraint term for the sparse representation framework, thereby leading to more robust tracking results.

(3) Through the accelerated proximal gradient (APG) method [31], the optimal solution (in matrix form) of the above constrained sparse tracking model is derived and can be quickly obtained within several iterations. Moreover, the matrix-form solution allows the candidate samples to be processed in parallel, which can further improve the calculation efficiency of the tracker.

The rest of this paper is organized as follows. In Section 2, the related background contents of the proposed tracker are reviewed. The detailed tracking implementations are presented in Section 3, and the insights of the proposed method are discussed in Section 4. In Section 5, experimental results and related analyses are demonstrated. Finally, the conclusion is given in Section 6.

## 2. Background Contents

To facilitate the presentation of the proposed tracker, in the following sections, we briefly review some basic contents, including the particle filter, the sparse representation-based tracking model (L1 tracker) and ELM.

### 2.1. Particle Filter

In this work, visual tracking can be considered as a Bayesian inference task in a Markov model with hidden state variables. Without knowing the concrete object observation possibility, the particle filter [32] could provide an object *a posteriori* estimation related to the Markov chain. The particle filter can be viewed as two steps: prediction and updating. We denote $s_{l}$ as the motion state of object at frame *l* and the observation set of object $Z_{l-1}=\{z_{1},z_{2},\cdots z_{l-1}\}$ from the first frame to the frame l−1. For the prediction stage, the object state $s_{l}$ can be predicted as:

(1) p(slslZl−1)Zl−1)=∫p(slslsl−1)sl−1)p(sl−1sl−1Zl−1)Zl−1)dsl−1

Given the observation of object $z_{l}$ at frame *l*, the observation possibility of $s_{l}$ can be updated as:

(2) p(slslZl)Zl)∝p(zlzlsl)p(slslZl−1)Zl−1)sl)p(slslZl−1)Zl−1)

The object motion state $s_{l}$ is composed of six affine transformation parameters $\left\{\theta_{1},\theta_{2},\theta_{3},\theta_{4},\theta_{5},\theta_{6}\right\}$. Here, $\left\{\theta_{1},\theta_{2}\right\}$ denote the 2D position changes, and $\left\{\theta_{3},\theta_{4},\theta_{5},\theta_{6}\right\}$ are the rotation angle, scale, aspect ratio and skew, respectively. In Equation (1), p(slslsl−1)sl−1) denotes the state transition probability, and it is often modeled by the Gaussian distribution: p(slslsl−1)sl−1)=N(sl;sl−1,Ω). Here, **Ω** is a diagonal matrix, and its diagonal elements are the variances of six affine transformation parameters.

(3) $$\text{Ω}=diag\left(\sigma_{\theta_{1}}^{2},\sigma_{\theta_{2}}^{2},\sigma_{\theta_{3}}^{2},\sigma_{\theta_{4}}^{2},\sigma_{\theta_{5}}^{2},\sigma_{\theta_{6}}^{2}\right)$$

In Equation (2), p(zlzlsl)sl) essentially reflects the likelihood of observing $z_{l}$ upon the state $s_{l}$. Finally, the optimal state ${\hat{s}}_{l}$ can be estimated via the maximum *a posterior* (MAP) estimation.

(4) s^l=argmaxslip(slisliZl)Zl)

### 2.2. Sparse Representation-Based Tracker

The appearance of an object under different viewpoints and illumination conditions is known to lie approximately in a low-dimensional subspace [12]. For visual tracking, this subspace consists of some target templates: $T^{\circ }=\left[t_{1}^{\circ },\ldots ,t_{n}^{\circ }\right]\in R{}^{d\times n}$. Here, *d* is the dimension of the template sample, and the object sample $y\in R{}^{d}$ can approximately lie in the linear span of $T^{\circ }$.

(5) $$y\approx T^{\circ }a_{T}=a_{1}t_{1}^{\circ }+a_{2}t_{2}^{\circ }+\cdots +a_{n}t_{n}^{\circ }$$

where $a_{T}=\left(a_{1},a_{2},\cdots ,a_{n}\right)\in R{}^{n}$ is the target coefficient vector of sample y in the target subspace. To account for the background noise and likely occlusion, the identity matrix set $I^{\circ }={\left[i_{1}^{\circ },i_{2}^{\circ },\cdots ,i_{d}^{\circ }\right]}^{T}\in R{}^{d\times d}$ is introduced into the linear representation, that is:

(6) $$y=\left[T^{\circ },I^{\circ },-I^{\circ }\right]{\left[a_{T},a_{I}\right]}^{T}=\mathrm{Da}$$

The space D consists of target templates and trivial ones, and D is used for constructing the sample data. $a_{I}=\left(e_{1},e_{2},\cdots ,e_{2d}\right)\in R{}^{2d}$ is the sparse trivial coefficient vector. Thus, the L1 tracker finds the construction of sample y in the space D by solving the following minimization problem.

(7) $$\min_{a}{∥y-\mathrm{Da}∥}_{2}^{2}+\lambda {∥a∥}_{1},\;\;s.t.\;\;\;a\geq 0$$

In the above problem, ${∥\cdot ∥}_{2}$ and ${∥\cdot ∥}_{1}$ denote the l2 norm and l1 norm, respectively. *λ* is the regularization parameter. Via the optimization techniques, the optimal solution $\hat{a}$ is obtained, and the corresponding likelihood of sample y belonging to the target is calculated by:

(8) $$\exp\left\{-{∥y-T^{\circ }{\hat{a}}_{T}∥}_{2}^{2}\right\}$$

Here, ${\hat{a}}_{T}$ is the target coefficient vector of the optimal solution $\hat{a}$ from Equation (7).

### 2.3. Extreme Learning Machine

The extreme learning machine presented by Huang *et al*. [25] is a novel and efficient machine learning technique for training single layer feed-forward networks (SLFNs). Suppose that SLFNs have *L* hidden nodes, and it can be represented as follows.

(9) $$f_{L}\left(x\right)=\sum_{j=1}^{L}G\left(w_{j},b_{j},x\right)\beta_{j}=\sum_{j=1}^{L}h_{j}\left(x\right)\beta_{j}$$

Here, $w_{j}$ is the input weight connecting the input layer to the *j*-th hidden node and $b_{j}$ is the bias of *j*-th hidden node. G(·) is the activation function, and $G(w_{j},b_{j},x)$ indicates the output vector of the *j*-th hidden node. $\beta_{j}$ is the output weight connecting the *j*-th hidden node to the output. Unlike the traditional understanding of neural networks, ELM theories [25,26] show that hidden neurons need not be adjusted. The corresponding implementation is the random hidden neurons, and its parameters (*i.e*., w and *b*) are randomly generated based on a continuous probability distribution. Additionally, the ELM network has both universal approximation capability and classification capability.

**Theorem 1**. *Universal approximation capability [33]: Given any bounded nonconstant piecewise continuous function as the activation function, if the SLFNs can approximate any target function* f(x) *via tuning the parameters of hidden neurons, then the sequence* ${\left\{h_{j}\left(x\right)\right\}}_{j=1}^{L}$ *can be randomly generated based on any continuous sampling distribution, and* $\lim_{L\to \infty }∥\sum_{j=1}^{L}h_{j}\left(x\right)\beta_{j}-f\left(x\right)∥=0$ *holds with probability one with the appropriate output weight **β***.

**Theorem 2**. *Classification capability [30]: Given any feature mapping* h(x), *if* h(x)β *is dense in* $C(R^{d})$ *or in* C(M), *where M is a compact set of* $R^{d}$, *then SLFNs with a random hidden layer mapping* h(x) *can separate arbitrary disjoint regions of any shapes in* $R^{d}$ *or M*.

Due to that ELM only computing the output weights analytically, it will have a faster learning speed. Meanwhile, experimental results show that ELM also has a good generalization in various applications [27,28,29,34,35,36]. The ELM algorithm is reviewed as follows.

Given a training dataset $\left\{X,T\right\}={\left\{x_{i},t_{i}\right\}}_{i=1}^{N}$, $x_{i}\in R{}^{d}$ is the *i*-th training data, and the corresponding label is $t_{i}\in \left\{0,1\right\}$. The ELM training is to obtain both the smallest norm of output weights and the smallest training error.

(10) $$\hat{\beta }=\underset{\beta }{\arg\min}\left\{{∥\beta ∥}_{2}^{2}+\frac{\mu }{2}{∥T-H\beta ∥}_{2}^{2}\right\}$$

where *μ* is a regularization parameter. H is the hidden layer output matrix, which is defined by:

(11) $$H={\left[\begin{array}{ccc} G(w_{1},b_{1},x_{1}) & \cdots & G(w_{L},b_{L},x_{1})\\ \vdots & \vdots & \vdots \\ G(w_{1},b_{1},x_{N}) & \cdots & G(w_{L},b_{L},x_{N}) \end{array}\right]}_{N\times L}$$

Via the gradient numerical method, the optimal solution of ELM training can be obtained.

(12) β=IIμμ+HTH−1HTT,ifL≤NHTIIμμ+HHT−1T,ifL>N

The learning efficiency and generalization performances of Equation (12) have been studied in [30].

## 3. Proposed Tracking Algorithm

**Figure 1:**
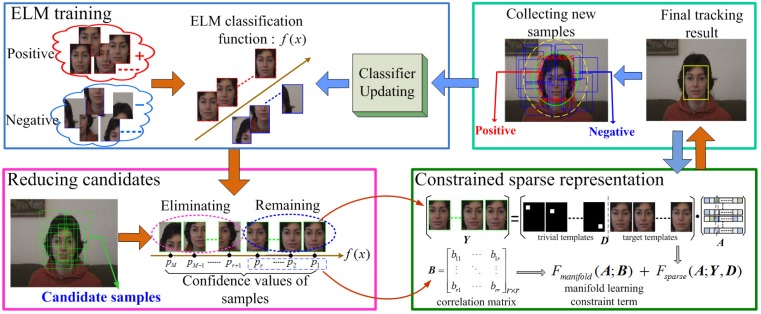
Flowchart of the proposed tracking algorithm.

To achieve a better sparsity tracker in terms of efficiency and robustness, we plan to exploit the ELM technique in the sparse visual tracking framework. The flowchart of the proposed method is shown in Figure 1. One can see that the ELM method firstly finds the optimal separated hyperplane between the target (positive) samples and background (negative) ones. Within the particle filter framework, a great deal of candidate particles are sampled for estimating the object state. To reduce the calculated amount of particle sparse representation, the trained ELM classification function is utilized for removing most of the particle samples related to the background. Then, the resultant confidence values of particle samples on the ELM classification function are viewed as the probabilities belonging to the target and used for establishing the manifold learning constraint term of the following sparse representation, which tends to improve the robustness of the tracker. Finally, the estimated tracking result is to update the target templates of the sparse representation, and some new positive and negative samples are collected for updating the ELM classifier online. Thus, the proposed tracker could adaptively handle the visual changes of the foreground and backgrounds during tracking, thereby leading to more robust tracking results.

### 3.1. ELM Training

To promote the tracking efficiency of the sparse representation-based tracker, the number of particles in the sparse representation should be reduced. In [13], a portion of candidate particles is removed via computing the reconstruction error in the target space. However, there still exist some particles relevant to the backgrounds, and the tracking speed is thus restricted to some extent. In this subsection, to make full use of the comparison information between the target and its backgrounds, a binary classifier is firstly trained for discarding most of the particle samples related to the background contents. The adopted classifier should have a low computational burden and a good discriminative capability. Compared to other techniques, the ELM model has a faster implementation and tends to achieve better generalization [30]. Thus, the ELM technique is preferred in visual tracking, and the detailed procedure is as follows.

#### 3.1.1. Generating Initial Training Samples

The object state in the first frame is often manually or automatically located. Supposing the initial center of the object is $J_{0}$, the initial positive (target) samples are randomly generated within a circular area defined by $∥J_{0}-J_{i}∥<\varphi$, where $J_{i}$ is the center of the *i*-th sampled patch. Meanwhile, the initial negative (background) samples are collected from an annular region defined by $\varphi <∥J_{0}-J_{j}∥<\varpi$ (*φ* and *ϖ* are inner and outer radii, respectively). Let $X=\left\{x_{1}^{p},\cdots ,x_{N_{p}}^{p},x_{1}^{n},\cdots ,x_{N_{n}}^{n}\right\}\in R{}^{N\times d}$ denote the sampled training dataset and $T=\left[1,\cdots ,1,0,\cdots ,0\right]\in R{}^{N}$ be the corresponding class labels. Here, $N_{p}$ and $N_{n}$ are the number of positive and negative samples, respectively.

#### 3.1.2. Classifier Training

Let the hidden node number of the ELM classifier be *L*. The input hidden parameters $w_{j}$ and $b_{j}$ (j=1,⋯,L) are randomly assigned based on a Gaussian distribution. Then, the hidden layer output matrix is computed via $H_{i}={\left\{h_{j}\left(x\right)\right\}}_{j=1}^{L}\left(i=1,\cdots ,N\right)$, where $h_{j}\left(x\right)=G\left(w_{j},b_{j},x\right)$ is the *j*-th hidden node output. For visual tracking, we only have the object state in the first frame and no other information in a video. Thus, the number of collected training samples is not sufficient. Meanwhile, for a good generalization, the hidden node number *L* is generally larger than the number of initial training data from the first frame. In this case, the output weights ***β*** of the ELM classifier are calculated by:

(13) β=HTIIμμ+HHT−1T

The corresponding ELM classification function is:

(14) f(x)=h(x)β=h(x)HTIIμμ+HHT−1T

Considering the truth that the number of initial training samples in the first frame is not sufficient, the trained classifier may not have good adaptability. To expand the training sample set, we uniformly collect L/2 positive and L/2 negative training samples from the initial five tracking frames and ensure that the total number of expanded training samples is larger than the hidden node number. Therefore, the new output weights ***β*** of the retrained ELM classifier are computed by:

(15) β=IIμμ+HTH−1HTT

For the subsequent tracking frames, the likelihoods belonging to the true object of particle samples are calculated via the following function.

(16) f(x)=h(x)β=h(x)IIμμ+HTH−1HTT

#### 3.1.3. Classifier Updating

During the tracking process, to deal with the appearance changes of both targets and environments, the tracker should update the ELM classifier online. The input hidden parameters (w and *b*) and hidden node number *L* are fixed and never changed. Thus, the updating of ELM is equal to updating the output weights ***β***. Simply, the ELM model can be retrained with the total training data (including the old data). However, this kind of updating needs to store more and more training samples, and retraining with a large number of samples is a time-consuming task. Under this condition, the online sequential technique [37] is exploited for updating the ELM model.

Suppose now that we already have some training samples ${ℵ}_{0}=\left\{X_{0},T_{0}\right\}={\left\{x_{i},t_{i}\right\}}_{i=1}^{N_{0}}$, and $N_{0}$ is the number of initial training data. According to the above subsection, the output weights $\beta_{0}$ are:

(17) β0=IIμμ+H0TH0−1H0TT0=P0−1H0TT0

Here, $H_{0}$ is the initial calculated hidden output matrix. Given $N_{1}$ new training data ${ℵ}_{1}=\left\{X_{1},T_{1}\right\}={\left\{x_{i},t_{i}\right\}}_{i=N_{0}+1}^{N_{0}+N_{1}}$, we can update the output weights as follows.

(18) $$\beta_{1}=P_{1}^{-1}{\left[H_{0},H_{1}\right]}^{T}\left[T_{0},T_{1}\right]$$

In Equation (18), P1=IIμμ+H0,H1TH0,H1=P0+H1TH1. With these equations, we can easily obtain the incremental updating expression of output weights ***β***.

(19) $$\beta_{1}=\beta_{0}+P_{1}^{-1}H_{1}^{T}\left(T_{1}-H_{1}\beta_{0}\right)$$

From the above derivation, one can see that the updating of the ELM model only considers computing the new training data, and the resultant output weights can obtain the same learning accuracy as the retrained holistic training using the whole training data.

### 3.2. Reducing Candidate Samples

This work treats the tracking as a Bayesian inference task with hidden state variables, and the particle filter technique [32] is utilized for providing the object *a posteriori* estimation. With the assumption that the object state transition probability follows the Gaussian distribution, *M* candidate particles are sampled to estimate the object state: $Y=\{y_{i}\in R{}^{d\times 1}\},\;i=1\ldots M$. For tracking accuracy, the number of candidate particles is often huge. In Section 3.1, the ELM classifier is well trained with respect to the target and background samples. Generally, the potential target particles have high ELM functional output values, and *vice versa*. Thus, with the trained ELM classification function f(·), those particles related to the background contents can be removed. Then, only a few potential particles are selected for the particle sparse representation. The detailed implementation is as follows.

The confidence value of one candidate particle $y_{i}$ is defined by $p_{i}$. With the same input hidden parameters (w and *b*) as the ELM training stage, the corresponding hidden node output of $y_{i}$ is calculated by $h\left(y_{i}\right)=G\left(w,b,y_{i}\right)$. Using the suitable output weights ***β***, we compute the confidence value $p_{i}$ by:

(20) $$p_{i}=f\left(y_{i}\right)=h\left(y_{i}\right)\beta$$

According to the confidence score value, the candidate particles are ranked in descending order: $\left\{p_{1},p_{2},\cdots ,p_{M}\right\}$. Here, $p_{i}>p_{i+1},\;\forall i$. The $p_{i}$ value essentially indicates the probability of particle $y_{i}$ belongs to object. We only choose a small number of high-ranking samples: $\left\{p_{1},p_{2},\cdots ,p_{r}\right\}\;\left(r<M\right)$, and the other candidate samples are discarded, thereby alleviating the calculation amount for the particle sparse representation. Here, we define the proportion parameter *ζ* as ζ=r/M, and the value of *ζ* controls the percentage number of the selected particles for the following sparse representation. The reducing candidate operation can lead to two advantages. One is to cut down the computational burden of the tracker. The other is that the ELM model is discriminative enough for separating the object from its backgrounds. Thus, it can be free from the background disturbance to some degree, which tends to achieve stabler solutions.

### 3.3. Constrained Sparse Representation

#### 3.3.1. Model Formulation

According to the manifold learning framework, two similar candidate samples should have similar coefficients in sparse representation [18]. To enforce this assumption on these particle samples, the following loss function is presented.

(21) $$F_{manifold}\left(A;B\right)=\sum_{ij}{∥a_{i}-a_{j}∥}^{2}B_{ij}$$

Here, $a_{i}$ and $a_{j}$ are the sparse coefficients of particle $y_{i}$ and particle $y_{j}$, respectively. $B_{ij}$ is the correlation coefficient, which indicates the similarity relation of two particles. If the particle $y_{i}$ is similar to particle $y_{j}$, the $B_{ij}$ is large; otherwise, the $B_{ij}$ is small. To further combine the ELM learning and sparse representation, we calculate the correlation coefficient with the resultant confidence values of particle samples on the ELM classification function.

(22) $$B_{ij}=\exp\left(-{∥p_{i}-p_{j}∥}^{2}\right)$$

With the calculated B, we define these expressions: $\Gamma_{i}=\sum_{j=1}^{r}B_{ij}$, $\hat{\text{Γ}}=diag\left(\Gamma_{1},\Gamma_{2},\cdots ,\Gamma_{r}\right)$, $L=\hat{\Gamma }-B$. Here, diag(·) denotes the diagonalizable matrix operator. The Equation (21) can be transformed as:

(23) $$\begin{array}{c} F_{manifold}\left(A;B\right)=\sum_{ij}{∥a_{i}-a_{j}∥}^{2}B_{ij}\\ =\sum_{i}{∥a_{i}∥}^{2}\Gamma_{i}+\sum_{j}{∥a_{j}∥}^{2}\Gamma_{j}-2\sum_{ij}a_{i}^{T}a_{j}B_{ij}=2\times \mathrm{Tr}\left(\mathrm{AL}A^{T}\right) \end{array}$$

where $\mathrm{Tr}\left(\cdot \right)$ is the trace of a matrix. As a new regularization item, $F_{manifold}\left(A;B\right)$ is introduced into the objective function of basic sparse representation Equation (7).

(24) $$\min_{A}{∥Y-\mathrm{DA}∥}_{2}^{2}+\lambda {∥A∥}_{1}+\gamma \mathrm{Tr}\left(\mathrm{AL}A^{T}\right),\;\;s.t.\;\;A\geq 0$$

Here, Equation (24) is a problem of constrained sparse representation, in which ELM learning is further combined with the particle sparse representation via a regularization term. Thus, the solution of Equation (24) can be robuster. In addition, the computing of the correlation matrix in the manifold learning term does not depend on the time-consuming KNN method, thereby contributing to the tracking efficiency.

#### 3.3.2. Model Solution

In this subsection, we aim to solve the problem of Equation (24). The indicated function ϕ(ω) is defined by:

(25) $$\phi \left(\omega \right)=\left\{\begin{array}{cc} 0 & \;\;\;\omega \geq 0\\ +\infty & \;\;\;\omega <0 \end{array}\right.$$

With the indicated function ϕ(ω), the minimization problem of Equation (24) is equivalent to:

(26) $$\min_{A}{∥Y-\mathrm{DA}∥}_{2}^{2}+\lambda A+\gamma \mathrm{Tr}\left(\mathrm{AL}A^{T}\right)+\phi \left(A\right)$$

Obviously, the object function of Equation (26) consists of two different parts: the first three terms are the differentiable smooth convex functions with a continuous gradient, and the last term is a non-smooth, but convex function. For solving this kind of optimization problem, the accelerated proximal gradient (APG) method could yield an O(11k2k2) residual from the optimal solution after *k* iterations [31]. For clear representation, we rewrite the object function of Equation (26) as:

(27) $$\left\{\begin{array}{c} F\left(A\right)={∥Y-\mathrm{DA}∥}_{2}^{2}+\lambda A+\gamma \mathrm{Tr}\left(\mathrm{AL}A^{T}\right)\\ G(A)=\phi (A) \end{array}\right.$$

With the APG method, we can calculate the sparse representation coefficients A via Algorithm 1. In Algorithm 1, $M_{k}$ is a temporary variable, and the gradient function $\nabla F\left(A\right)$ is:

(28) $$\nabla F\left(A\right)=2D^{T}\left(\mathrm{DA}-Y\right)+\lambda 1_{D}1_{Y}^{T}+\gamma A\left(L^{T}+L\right)$$

Here, $1_{D}\in R^{N_{d}}$ and $1_{Y}\in R^{r}$ are the column vectors whose entries are all ones. $N_{d}$ is the number of templates in sparse representation space, and *r* is the number of selected particles for sparse representation. The key in the APG method is to solve the optimization problem of Step 2. Additionally, with the indicated function ϕ(ω), this optimization problem has the following closed form solution.

(29) $$A_{k+1}=\max\left(0,\;\;M_{k+1}-\frac{\nabla F(M_{k+1})}{\xi }\right)$$

With the above closed form solution, the optimal solution of the constrained sparse tracking model can be achieved within several iterations. It should be noted that the optimal solution is derived in matrix form. Thus, it allows the particle samples to be calculated in parallel, which can further improve the tracking efficiency of the proposed tracker. After calculating the optimal sparse representation coefficients $\hat{A}$, the probability of one candidate sample belongs to object is computed via Equation (8), and the final tracking result can be estimated via the MAP estimation.
**Algorithm 1:** Solving sparse representation coefficients A.
**Input:** The templates set D, the candidate samples Y and the Lipschitz constant *ξ*.1: The initial value of optimal solution: $A_{0}=A_{-1}=0$, iteration parameter: $\varsigma_{0}=\varsigma_{-1}=1$.2: Calculating the following expressions, k=0,1,…, not until convergence. $\left\{\begin{array}{c} M_{k+1}=A_{k}+\frac{\varsigma_{k-1}-1}{\varsigma_{k}}\left(A_{k}-A_{k-1}\right)\\ \varsigma_{k+1}=\frac{1+\sqrt{1+4\varsigma_{k}^{2}}}{2}\\ A_{k+1}=\arg\min_{A}\frac{\xi }{2}{∥A-M_{k+1}+\frac{\nabla F(M_{k+1})}{\xi }∥}_{2}^{2}+G\left(A\right) \end{array}\right.$**Output:** The calculated sparse representation coefficients $\hat{A}$.

### 3.4. Template Updating Scheme

To adapt to the visual changes of the foreground and its backgrounds during the tracking process, the tracker needs to update the target templates of the sparse representation space and select new training samples for updating the ELM classifier. Similar to [18], we update the target templates with the tracking result. However, the confidence value of the updated template on the ELM classification function f(·) should be larger than a threshold value *τ*. Under this condition, we can prevent some inappropriate updating samples from degrading the tracker. In addition, the tracking result from sparse representation is treated as the new object center. The same strategy as adopted in the first frame is applied for selecting new positive and negative training samples. One can see that this template updating scheme considers two factors from the ELM learning and sparse representation. Therefore, from updating the tracker point of view, the effects of single classification error or sparse reconstruction error can be decreased via combining these two different models.

## 4. Discussion

### 4.1. Preference of the ELM Technique in Visual Tracking

In this paper, ELM is applied for modeling the comparison information between the foreground and backgrounds. As is known to all, SVM is also one of the successful classifier techniques and has been applied in many applications. For visual tracking, the computational burden should not be high. However, as analyzed by Huang *et al.* [26], the training of SVM is a quadratic programming problem, and thus, it requires high computational cost. In contrast, the parameters of the ELM hidden layer need not be adjusted and could be independent of the training data. Hence, the ELM model only computes the output weights analytically, and it has a much faster learning speed and lower computational complexity than SVM. For tracking efficiency, the ELM model is preferred in visual tracking.

Instead of the inequality constraints used in the basic SVM, equality constraints are adopted in the least square SVM (LS-SVM), and the resulting solution is closed form. Owing to this, LS-SVM has been used in some real-time applications. For SVM/LS-SVM, its feature-mapping function ψ(x) is often implicit and unknown. Therefore, in theory [38], the universal approximation capability of LS-SVM/SVM has not been proven. From the optimization point of view, SVM/LS-SVM suffers from the curse of local minima. However, it has been proven that the ELM model has a universal approximation capability [33]. Thus, we do not encounter any local minima issues when applying the ELM technique. Additionally, in [30], experiments also validate that ELM tends to be superior in terms of generalization compared to LS-SVM.

Furthermore, in our experiments (see Section 5.4), we compared the tracking performances between using ELM and SVM/LS-SVM, which experimentally demonstrate that the ELM model tends to be preferred in visual tracking.

### 4.2. Insights about the Combination of ELM and Sparse Representation

To the best of our knowledge, this is the first attempt to combine the extreme learning machine and sparse representation in the visual tracking field. With the advantages of ELM and sparse representation, in [34], a hybrid approach combining ELM and sparse representation classification (SRC) is also presented for image classification and reports some good results. However, the existing hybrid method is just a simple combination of ELM and SRC. It should be noted that our work is not the simple cascading combination of two different techniques. In our work, this further combination can be detailed by two aspects. One is that most of the candidate samples with low confidence values are relevant to the backgrounds and then may be abandoned. With this operation, the resulting calculation cost of particle sparse representation and the disturbance from background candidate samples are both alleviated.

The other one is that the confidence value of the particles can reflect the similarity degree among these particles. Intuitively, two similar particles should have approximate confidence values on the ELM classification function f(·). Based on this rationale, we compute the correlation matrix B with the resultant confidence values of the particles. Additionally, under the manifold learning framework, a new regularization item Equation (23) with respect to the sparse representation coefficients is built. Unlike the simple hybrid method [34], the proposed constrained sparse representation model fully considers the results from ELM learning and sparse representation, thereby tending to achieve more robust performances.

## 5. Experimental Results and Analysis

### 5.1. Experimental Setup

The proposed tracker is implemented on MATLAB R2010b, Intel(R) B960, 2.20-GHz CPU, 2 GB RAM, Win7 x86 system, Lenovo, beijing, China. The raw pixel intensity is used as the image feature representation of the input samples. We resize the obtained image sample to a 32×32 pixel patch. As a trade-off between effectiveness and speed, 600 particle samples are generated; the iteration number *k* of Algorithm 1 is set as 5, and our tracker is incrementally updated every 5 frames. For the ELM classifier, the activation function G(·) is set as the sigmoid function, and in the initial five tracking frames, we uniformly select L/2 positive samples and L/2 negative ones to train the initial ELM classifier. Besides, 50 positive samples and 50 negative samples are collected for online updating of the ELM classifier. As for sparse representation, the number of target template spaces is set to be 20.

In this paper, based on empirical results, the ELM hidden node number *L* is set as 1000, and the regularization parameters are set as μ=0.1, λ=0.1 and γ=0.1. In addition, the proportion parameter *ζ* is set as 0.25, and the confidence score threshold *τ* is chosen to be 0.6 empirically. For setting the above parameters, there is not a best possible way. Therefore, they are to be determined under a trial-and-error procedure. The settings and effects of these parameters are specifically discussed in Section 5.3.

### 5.2. Traditional Evaluation

To evaluate whether the proposed tracker can handle the tracking challenges well, including illumination variation, pose change, partial occlusion, background clutter, motion blur, scale change, object rotation, and so on, we have utilized 14 representative video sequences in our work. The differences between these adopted tracking sequences, and their corresponding challenging factors are illustrated in Table 1. It can be seen that these video sequences contain different numbers of frames, and different tracked objects (e.g., face, car, body, *etc*.) undergo different motion states (e.g., occlusion, abrupt motion, rotation, *etc*.).

It should be noted that the used video sequences are mainly from several published papers [1,18,21,39,40]. Our proposed tracker is compared to six state-of-the-art tracking methods, which are also discussed in these published papers. They are referred to as the L1 tracker [12], the L1APG tracker [14], the kernel SVM (K-SVM) tracker [21], the fragments-based (Frag) tracker [41], the compressive tracker (CT) [23] and the multiple instance tracker (MIL) [24]. For a fair evaluation, we utilize the source codes provided by the authors and the same ground truth data. Besides, for these video sequences, the same initial object position states are applied for all of the trackers.

As a whole, the obtained results of compared trackers in this work are consistent with those in these published papers [1,18,21,39,40]. However, for these compared trackers, some random factors are involved, such as the random sampling to generate candidate samples (including L1APG, L1, K-SVM and our methods), the random projection for feature sampling in the CT method and other slight randomness. Here, we run them five times and report the best results in this paper.

**Table 1:** The captions of the video sequences for the tracker evaluation.

No.	Image Sequence	No. of Frames	Challenging Factors
1	Car4	659	illumination variation, background noise, scale change
2	Car11	393	illumination variation, background clutter, scale change
3	Singer1	321	illumination variation, scale change
4	Caviar1	382	partial occlusion, scale change
5	Caviar2	500	partial occlusion, scale change
6	Occlusion1	898	partial occlusion
7	Occlusion2	819	partial occlusion, object rotation
8	Mhyang	1490	illumination variation, pose variation
9	Girl	500	pose variation, partial occlusion, scale change
10	DavidIndoor	462	illumination variation, pose variation, object rotation
11	Dudek	1145	partial occlusion, pose variation, scale change
12	Dog1	1350	large scale change, object rotation
13	CarScale	252	background clutter, scale change
14	boy	602	abrupt motion, motion blur

#### 5.2.1. Quantitative Comparisons

In this subsection, the center location error (CLE), VOC [46] overlap rate (VOR) and frame per seconds (Fps) are used to evaluate the performances of each tracker on all of the challenging sequences. Here, the CLE measures the Euclidean distance between the centers of the tracker and the ground truth bounding box. Obviously, the CLE value of a good tracker should be small. The average CLEs on the 14 sequences are shown in Table 2. Besides, the VOR is used to describe the overall performance of each tracker, and it is defined with the ground truth bounding box $R_{G}$ and the estimated bounding box $R_{T}$.

(30) $$\mathrm{VOR}=\frac{\mathrm{area}(R_{T}\cap R_{G})}{\mathrm{area}(R_{T}\cup R_{G})}$$

If the VOR is greater than a predefined threshold, the frame is successfully tracked. Table 2 also reports the average VORs on the 14 video sequences. For the computational loads, we show the average Fps of each tracker and their code implementation software in Table 3. Generally, the high Fps indicates a faster tracking efficiency.

From the experimental results, one can see that the proposed tracker outperforms the other methods in terms of CLE and VOR, which validates the effectiveness of our tracker. Compared to the other three classic tracking algorithms (*i.e*., CT, MIL and Frag), the sparse representation-based trackers have achieved better results. However, the traditional L1 tracker suffers from the low efficiency issue. By using the minimum error bounded particle resampling strategy [13] and the APG solving method [14], the latter L1APG tracker has made progress in solving the time-consuming problem, but with some loss of tracking accuracy in some videos (e.g., Car4, Caviar2, *etc*.). The possible reason is that some potential candidates may also have large reconstruction errors in the target template space and then be discarded. Therefore, the resultant tracking accuracy tends to be reduced. Unlike the L1APG trackers, the proposed tracking method has achieved both superior performances on tracking efficiency and robustness. This can be attributed to the following aspects. (1) Using the comparison information between the target and its surroundings, the ELM classifier is trained quickly for removing most of the particles related to the background. Due to the ELM model being discriminative enough for distinguishing the target and backgrounds, the few best potential candidates can be retained for the later sparse representation task, which will contribute to both the tracking speed and robustness; (2) The proposed tracking method is based on a novel constrained sparse representation model, in which the ELM learning results are embedded as a manifold learning constraint term. Thus, the resulting solution will be more effective than the traditional sparsity-based trackers; (3) The APG numerical method is also applied for driving the optimal solution (in matrix form) of the constrained sparse tracking model. The matrix-form formulation allows the particle samples to be calculated in parallel rather than one by one, as most sparsity-based trackers do. In addition, among the other trackers, the K-SVM method has obtained the best performance. Due to the training of SVM needing to solve a quadratic programming problem, its tracking speed is thus lower than our tracker. Additionally, benefiting from the combination of constrained sparse representation and ELM learning, the proposed tracker also achieves a better tracking accuracy than the K-SVM method.

**Table 2:** Performance comparisons in terms of center location error (CLE) (in pixels) and VOC overlap rate (VOR) (%). The best results are shown in bold and red fonts. MIL, multiple instance tracker; CT, compressive tracker; Frag, fragments-based tracker; APG, accelerated proximal gradient.

Sequence	CT		MIL		K-SVM		Frag		L1		L1APG		Proposed	
	CLE	VOR	CLE	VOR	CLE	VOR	CLE	VOR	CLE	VOR	CLE	VOR	CLE	VOR
Car4	63.5	0.21	60.1	0.34	17.8	0.48	179	0.22	4.11	0.84	6.63	0.82	**3.54**	**0.89**
Car11	16.7	0.44	43.5	0.17	17.2	0.43	63.9	0.09	33.3	0.43	1.89	0.79	**1.46**	**0.85**
Singer1	16.1	0.34	15.2	0.34	31.6	0.38	22.1	0.34	4.57	0.71	4.43	0.69	**4.11**	**0.83**
Caviar1	16.7	0.52	48.2	0.26	3.79	0.69	5.53	0.68	119	0.28	56.4	0.29	**1.95**	**0.82**
Caviar2	66.3	0.29	69.8	0.25	5.62	0.71	5.64	0.56	3.34	0.79	62.1	0.35	**2.34**	**0.85**
Occlusion1	16.7	0.75	32.3	0.59	9.63	0.81	**5.62**	**0.89**	6.51	0.87	10.7	0.76	5.95	0.85
Occlusion2	17.3	0.58	14.1	0.61	7.94	0.65	15.5	0.61	11.1	0.67	10.4	0.72	**4.32**	**0.82**
Mhyang	13.3	0.61	20.3	0.51	5.12	**0.81**	12.5	0.65	3.45	0.77	5.27	0.72	**3.25**	0.79
Girl	18.8	0.31	13.7	0.41	**3.23**	0.71	20.6	0.46	3.34	0.68	3.54	0.66	3.26	**0.71**
DavidIndoor	15.7	0.45	18.3	0.43	6.25	0.54	82.1	0.18	18.9	0.45	21.5	0.36	**5.41**	**0.61**
Dudek	26.6	0.65	17.8	**0.71**	28.9	0.66	82.1	0.54	23.2	0.68	64.1	0.52	**12.5**	0.69
Dog1	6.96	0.54	7.82	0.54	6.26	0.66	12.1	0.55	3.75	0.66	3.41	0.74	**3.18**	**0.83**
CarScale	26.1	0.44	33.1	0.42	44.9	0.49	19.5	0.44	81.5	0.44	68.5	0.53	**12.3**	**0.79**
boy	9.03	0.51	12.8	0.51	2.64	0.72	40.5	0.39	6.98	0.74	7.21	0.77	**2.33**	**0.84**
Average	23.6	0.47	29.1	0.44	13.6	0.62	40.5	0.47	23.1	0.65	23.3	0.62	**4.71**	**0.80**

**Table 3:** Speeds and implementations of the tracking methods. Fps, frames per second.

Tracker	CT	MIL	K-SVM	Frag	L1	L1APG	Proposed
Average Fps	68	34	2	4	0.6	2.5	7
Software	MATLAB + C	C	C	C	MATLAB + C	MATLAB + C	MATLAB

#### 5.2.2. Tracking Efficiency Analysis

Generally, the computational burden of the tracking method affects its tracking efficiency. Thus, in this subsection, we first present some computational burden analysis contents of the compared methods and our proposed tracker. In our work, we exploit six compared tracking methods, including the CT, MIL, K-SVM, Frag, L1 and L1APG trackers.

As for the CT method, the compressive sensing makes its image feature extraction very efficient. Thus, the computational burden of the CT method is mainly from the naive Bayes classifier training and updating. In the MIL tracker, its computational cost is to compute the Haar-like features of the image sample and to train and update the multiple classifiers using the boosting technique. The kernel SVM model is applied in the K-SVM tracker. As is known to all, the training of the SVM is a quadratic programming problem and turns out to be a heavy computational burden for the tracker. For the Frag tracker, the calculation of vote maps for each fragment is the main computational task. Besides, it should be noted that tracking large targets has the same computational cost as tracking small ones due to the adopted integral histogram method in the Frag tracker. Both the L1 and L1APG trackers are sparsity tracking methods. In the L1 tracker, hundreds of L1-norm related minimization problems need to be solved, and the number of particles controls the total computational cost. By using the minimal error bounding strategy and an efficient numerical method, L1APG has reduced the computation load for the L1 tracker.

As for our proposed method, the tracking framework mainly consists of two steps. Specifically, ELM is employed as a binary classifier for selecting potential object particle samples, and then, sparse representation is used to determine the final candidate samples. Here, we further discuss the computational burdens or complexities of the above two steps.

(1) As for ELM classifier training, the total computational burden is composed of two parts. One is the initial training stage, and the other one is online sequential updating. In our work, Equation (15) is the initial training equation, and its main computational cost is to compute the matrix inversion, where $H^{T}H\in R^{L\times L}$ is used. Generally, the hidden nodes’ output matrix H is of full column rank. Then, we can apply the singular value decomposition to obtain the ELM solution. From the matrix computation theory [42], the resulting computational complexity is $O\left(dL^{2}N\right)$. Here, *d* is the feature dimension of training samples, *L* is the hidden nodes of the ELM classifier and *N* is the number of initial training samples. On the other hand, Equation (19) is the online sequential updating equation of ELM. Here, we only compute the new training data, and by using the above-mentioned numerical method, we can obtain the computational complexity $O\left(dLN_{1}^{2}\right)$, where $N_{1}$ is the number of new training data.

(2) As for solving the constrained sparse representation, the computational complexity is dominated by the sub-optimization problem of Step 2 in Algorithm 1. In our work, the closed form solution of this sub-optimization is Equation (29), and its computational burden is from computing the gradient of function F(A) (*i.e*., Equation (28). Thus, we can easily compute the per-frame complexity of constrained sparse representation to be O(kdr). Here, *k* is the inner iteration number, *d* is the feature dimension of the particles samples and *r* is the number of selected particle samples for sparse representation.

In addition, the code implementation software has an effect on the tracking efficiency. As far as we know, there are three basic code implementation methods for the existing trackers. Specifically, they are the MATLAB, C (C/C++) and MATLAB + C programs. In our work, the software platforms for all of the trackers are not same. However, to give a fair evaluation, we run the compared trackers using the codes and platforms provided by their authors. Therefore, in Table 3, we only show the tracking speeds on their corresponding software platforms. It can be seen that our proposed method is the only tracking algorithm that only depends on the MATLAB platform, and obtains seven frames per second (Fps).

#### 5.2.3. Qualitative Evaluation

This subsection illustrates some tracking results of the proposed tracker and other methods on 14 challenging video sequences. The challenges include illumination changes, background clutter, occlusions, pose variations, scale and scene changes, and so on.

(1) Illumination and Scale Changes

**Figure 2:**
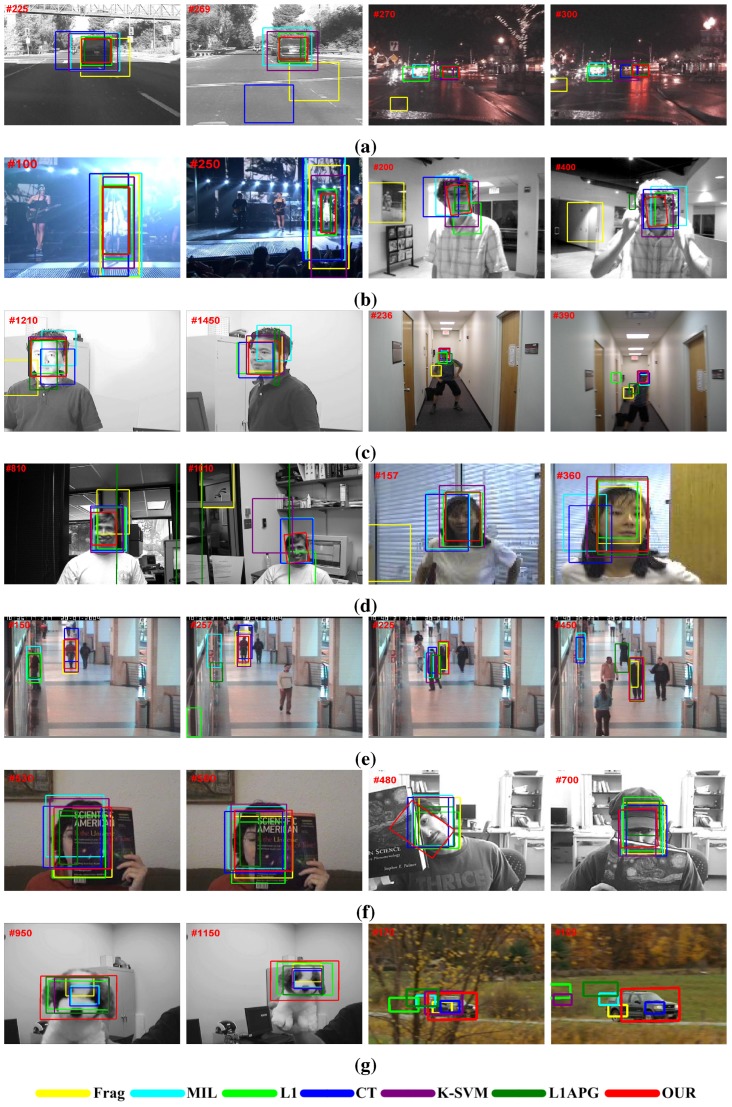
Representative tracking results on fourteen challenging sequences : (**a**) Car4 and Car11; (**b**) Singer1 and DavidIndoor; (**c**) Mhyang and boy; (**d**) Dudek and Girl; (**e**) Caviar1 and Caviar2; (**f**) Occlusion1 and Occlusion2; (**g**) Dog1 and CarScale.

In Figure 2a,b, some representative tracking results of the Car4, Car11 and Singer1 sequences are illustrated. In theses videos, the appearance of an object undergoes serious illumination and scale variations. The Frag, MIL, K-SVM and CT trackers could not simultaneously deal well with the illumination disturbance, as well as the scale changes. The accumulating tracking errors finally lead to the drifting problem (see Car4 #269, Car11 #300 and Singer1 #250). In contrast, the sparsity-based trackers could account for the background noise (including illumination variations) using the trivial templates and obtain robust tracking results. It is noted that, by using an effective occlusion detection, the L1APG tracker performs more robustly than the L1 method in the Car11 sequence (see Car11 #270). Moreover, the proposed tracker utilizes the ELM technique for quickly modeling the object and background contents, which can further boost the discriminative capability for separating the object from its surroundings. Therefore, the final tracking results of our method are more accurate than other sparsity-based trackers.

(2) Rotation and Pose Variations

Some tracking results of DavidIndoor, Mhyang, boy, Dudek and Girl are shown Figure 2b–d. The objects of five sequences are all moving faces, which suffer from rotation and pose variations, illumination changes, abrupt motion and partial occlusion. When dealing with the background noise and illumination changes, the performances of sparsity-based trackers are very robust (see Mhyang #1210, Girl #157). However, when serious pose variation (see DavidIndoor #200, Mhyang #1450, Girl #360) or motion blur (see boy #390) occurs, the trivial templates could not account well for the nonlinear changes of the object appearance. In this case, the online-trained ELM classifier of our tracker can deal with these appearance changes from pose variation or fast motion. In addition, via solving the constrained sparse tracking model (Equation (26)), the proposed tracker can decrease the tracking errors from the sparse reconstruction or binary classification, which tends to have more robust results than the traditional sparsity or classification tracking method.

(3) Heavy Occlusions

Occlusion is a big problem for visual tracking. The occlusion part will destroy the holistic appearance of the target. As shown in Figure 2e,f, we test four sequences (Caviar1, Caviar2, Occlusion1 and Occlusion2), which have severe occlusions. From these tracking results, one can see that only our tracker could keep track of the object through the entire sequence. Meanwhile, the target undergoes scale change or object rotation, and our tracker can accurately lock on the object all of the time (see Caviar1 #150, Occlusion2 #480). For addressing the occlusion issue, the robustness of our tracker benefits from two aspects. One is that the adopted ELM classifier can resist the occlusion disturbance via reducing the background candidate samples. The other is that the updating sample scheme avoids degrading the tracking model by rejecting inappropriate updating samples.

(4) Large-Scale Change and Background Clutter

Figure 2g demonstrates some tracking results on two sequences (Dog1 and CarScale) with large-scale changes and background clutter. In the Dog1 sequence, most trackers do not adapt to large-scale changes (e.g., Dog1 #950). The proposed method could follow the changing state for the entire sequence owing to the effective ELM updating in the presented tracking framework. For CarScale, the K-SVM could deal with the scale changes. However, it can not handle the background clutter from the tree branch well (see CarScale #170). In contrast, our tracker performs better in these conditions, which can be attributed to effective combining of the discriminative capability (*i.e*., separating the object from its backgrounds) of the ELM model and the generative function (*i.e*., accounting for the background noise) of the sparse presentation.

### 5.3. Effects of Key Parameters

In this subsection, we investigate the settings and effects of the key parameters in our work. Through using all of the video sequences, the average tracking scores (*i.e.*, VOR) and speeds (*i.e*., frames per second, Fps) are reported. To plot these two criteria data in one figure, the tracking speeds with varied parameters are rescaled by Fps/7. Here, 7 denotes the tracking speed of the final proposed tracking method (reported in Table 3). The key parameters of our work are shown in the following Table 4, and their setting and effects for the tracker are discussed as follows.

**Table 4:** The descriptions of the key parameters.

Chapter Content	Name List
3.1 ELM Classifier Training	ELM hidden nodes number: *L*
	Regularization parameter: *μ*
3.2 Reducing Candidate Samples	Proportion parameter: *ζ*
3.3 Constrained Sparse Representation	Regularization parameters: *λ* and *γ*
3.4 Template Updating Scheme	Updating threshold parameter: *τ*

#### 5.3.1. ELM Hidden Nodes Number: *L*

**Figure 3:**
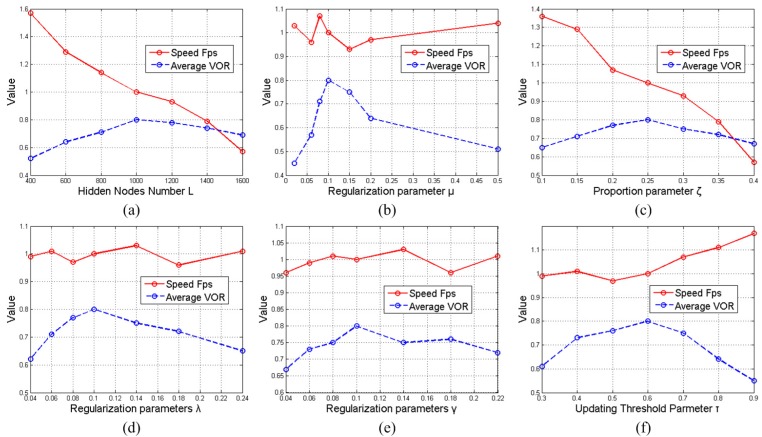
The effects of parameters: (**a**) *L*, (**b**) *μ*, (**c**) *ζ*, (**d**) *λ*, (**e**) *γ*, (**f**) *τ*.

For the ELM model, the hidden node number *L* is a very important parameter, which represents the Vapnik–Chervonenkis dimension of the ELM classifier. If the number *L* is too large, the trained separate hyperplane will be too complicated, which makes the tracker unable to adapt to the visual changes of the object and backgrounds during tracking. Additionally, if the number *L* is too small, the ELM classifier will have a poor discriminative capability, and thus, the proposed tracker cannot distinguish the object from its backgrounds. Figure 3a illustrates the tracking accuracies and speeds with different *L* numbers. One can see that the proposed tracker could achieve a satisfying result when L=1000. Therefore, we set the ELM hidden node number *L* to be 1000 in the proposed tracking method.

#### 5.3.2. Regularization Parameter: *μ*

In our work, the regularization parameter *μ* is another important factor for the ELM classifier, which provides a tradeoff between the norm of the ELM output weights and the smallest training error. Generally, if the value of *μ* is too large, the trained ELM framework would pay less attention to the norm of output weights, which makes the binary classifier unstable. The other way around, if the value of *μ* is too small, the ELM classifier could not take full advantage of the *a priori* knowledge, thereby leading to poor classification capability. In Figure 3b, the tracking performances with different *μ* values are shown. Empirical results demonstrate that the proposed method performs best when the value of *μ* is 0.1. Thus, we choose μ=0.1 as the default parameter of our tracker.

#### 5.3.3. Proportion Parameter: *ζ*

As for the proposed method, the ELM model is applied for selecting potential object particle samples for the following constrained sparse representation. The proportion parameter *ζ* is defined by ζ=r/M. Here, *r* is the number of selected particle samples and *M* is the total number of particle samples. If the value of *ζ* is too large, most of the particle samples will be kept. Hence, the function of the ELM framework will be lost, which cause the tracker to be not robust in some challenging cases (such as background clutter, motion blur). Meanwhile, the resultant total computational burden will be heavy. Additionally, if the value of *ζ* is too small, some potential object samples may be discarded by the ELM classifier, and thus, the final tracking accuracy will not be high. In Figure 3c, the tracking accuracies and speeds with different *ζ* values are reported, from which one can see that our tracker can achieve a satisfying result when ζ=0.25.

#### 5.3.4. Regularization Parameters: *λ* and *γ*

The regularization parameters *λ* and *γ* are related to the constrained sparse representation. Specifically, the value of *λ* controls the sparsity level of our sparse representation model, and the value of *γ* represents the enforcement of manifold learning in sparse representation.

For setting parameter *λ*, if the value of *λ* is too small, many templates (including trivial templates) will be maintained, and thus, the proposed tracker is not able to achieve robust performance owing to that it can not model the random background noises. If the value of *λ* is too large, the sparsity will be over-emphasized. Therefore, the final tracker cannot adapt to the dynamic diversity of particle samples.

For setting parameter *γ*, if the value of *γ* is too large, the manifold learning in sparse representation is also over-emphasized, thereby causing the tracker to not maintain the variety of particle samples. On the other hand, if *γ* is too small, the sparse representation is not able to make full use of the correlation information among the particle samples.

It can be seen from Figure 3d,e, when we choose λ=γ=0.1, the proposed tracking algorithm achieves the best performance.

#### 5.3.5. Updating Threshold Parameter: *τ*

In our work, the parameter *τ* controls the update scheme in our tracker. Generally speaking, if the value of *τ* is too large, it is difficult to select enough updating templates. Under this condition, the proposed tracker can not adapt well to the appearance changes of the object and backgrounds during tracking. Additionally, if *τ* is too small, some poor updating samples may be selected, and thus, the tracker will suffer from many unexpected noises and cannot obtain robust tracking results. From Figure 3f, we can see that the proposed tracker achieves good performance when the value of *τ* is around 0.6. Thus, we regard τ=0.6 as the default updating threshold.

### 5.4. Self-Comparisons

#### 5.4.1. Comparisons between ELM and SVM/LS-SVM

In this subsection, by using SVM/LS-SVM, we present two self-compared tracking methods, which can validate the preference of the ELM model in visual tracking. To give a fair evaluation, we only replace ELM with Gaussian kernel SVM/LS-SVM, and the following constrained sparse representation (CSR) model is not changed. Here, LS-SVM is one variant of the SVM technique, and it has been used in some existing trackers due to its closed form solution. It should be noted that the implementations of the above comparisons utilize the same parameter settings, and the software packages of SVM and LS-SVM are from the Lib-SVMlab [43] and LS-SVMlab [44].

**Figure 4:**
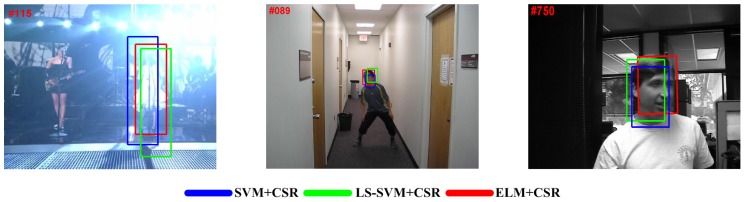
Some representative tracking results on three video sequences: Singer1, boy and Dudek (from left to right).

In Figure 4, some representative tracking results are illustrated. The utilized video sequences contain most of the tracking challenges, including illumination variation, pose and scale changes, partial occlusion, background clutter, motion blur, and so on. Besides, Table 5 also shows the compared details in terms of VOC overlap rate (VOR) and frames per second (Fps) tested on 14 challenging video sequences.

From these above comparisons, one can see that our tracker (ELM + CSR) performs better than the other two trackers in the field of tracking accuracy and efficiency, which experimentally demonstrate that the ELM model tends to be preferred in the visual tracking task.

**Table 5:** Comparisons between ELM and SVM/LS-SVM in terms of VOR (%) and Fps. CSR, constrained sparse representation.

Sequence	ELM + CSR	SVM + CSR	LS-SVM + CSR
Car4	**0.89**	0.78	0.76
Car11	**0.85**	0.76	0.74
Singer1	**0.83**	0.65	0.69
Caviar1	0.82	0.81	**0.85**
Caviar2	**0.85**	0.75	0.73
Occlusion1	**0.85**	0.56	0.51
Occlusion2	**0.82**	0.57	0.53
Mhyang	**0.79**	0.71	0.73
Girl	**0.71**	0.54	0.42
DavidIndoor	**0.61**	0.56	0.59
Dudek	**0.69**	0.63	0.61
Dog1	**0.83**	0.74	0.76
CarScale	**0.79**	0.75	0.71
boy	**0.84**	0.76	0.73
Average	**0.80**	0.68	0.67
Fps	**7**	1	6

#### 5.4.2. Contribution Verification of “ELM + Constrained Sparse Representation”

In our work, the ELM binary classifier is employed for selecting potential object particle samples and removing most of the background particle samples; and the constrained sparse representation (CSR) determines the final candidate sample. To investigate the contributions of the above two components (*i.e*., ELM and CSR) in our proposed tracker, in this subsection, we implemented two other trackers: Tracker1 and Tracker2.

For Tracker1, we take out the ELM binary classifier and only apply the constrained sparse representation in tracking. Here, the K-nearest neighbor (KNN) method [45] is exploited to compute the manifold learning term of sparse representation. To give a fair evaluation, the other parameter settings are not changed.

For Tracker2, we only utilize the ELM technique without CSR for locating the object, and the other settings are not changed. In our work, the output values of particle samples on the ELM classification function can be treated as the probabilities belonging to the target. Thus, based on the the maximum *a posteriori* (MAP) estimation, we can directly determine the object candidate by the ELM model.

**Figure 5:**
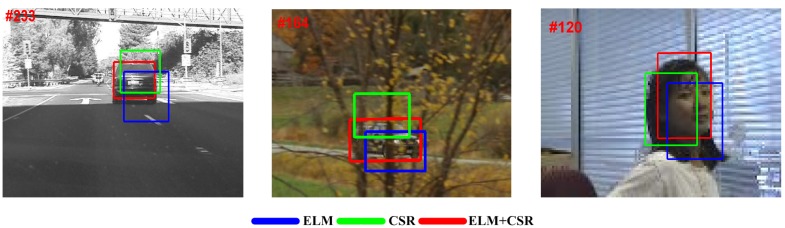
Some tracking results for self-comparisons on three video sequences: Car4, CarScale, Girl (from left to right).

These two self-compared methods are tested over all fourteen video sequences, and we calculate the average VOC overlap rate (VOR) and average tracking speed, which are reported in Table 6. On the other hand, in terms of qualitative evaluation, Figure 5 illustrates some representative tracking results.

Obviously, from the comparison results, our tracker (ELM + CSR) has achieved more accurate tracking results than the other two self-compared methods. Specifically, without the action of the ELM classifier, Tracker1 (CSR) suffers from poor tracking efficiency (Fps = 2). Besides, Tracker1 performs badly in some challenging cases (e.g., background clutter, motion blur) owing to its weak discriminative capability. As for Tracker2 (ELM), without the following sparse representation, it is more efficient than our method. However, from the comparison results, Tracker2 does not perform well in some sequences (e.g., Car4, Girl). The possible reason is that the ELM classifier is weak in modeling the random noise appearing in the object-self area. The proposed tracker can address this issue by introducing the constrained sparse representation and obtain a more robust and efficient tracking.

**Table 6:** Self-comparisons in terms of VOR (%) and frames per second (Fps).

Sequence	ELM + CSR	CSR	ELM
Car4	0.89	0.76	0.56
Car11	0.85	0.69	0.71
Singer1	0.83	0.61	0.54
Caviar1	0.82	0.54	0.69
Caviar2	0.85	0.66	0.72
Occlusion1	0.85	0.71	0.69
Occlusion2	0.82	0.62	0.61
Mhyang	0.79	0.69	0.75
Girl	0.71	0.57	0.63
DavidIndoor	0.61	0.58	0.55
Dudek	0.69	0.57	0.66
Dog1	0.83	0.71	0.73
CarScale	0.79	0.59	0.69
boy	0.84	0.72	0.75
Average	0.80	0.64	0.66
Fps	7	2	10

### 5.5. Benchmark Evaluation

**Figure 6:**
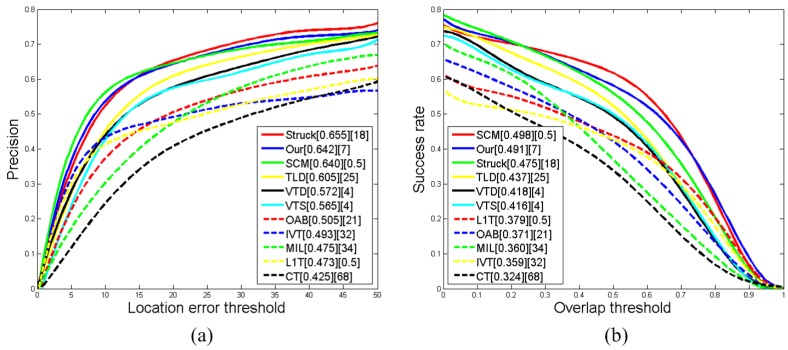
The performance score for each tracker is shown in the legend. (**a**) The precision plots of CLE, and the score is the precision at the threshold of 20 pixels; (**b**) the success plots of VOR, and the score is the AUC value. The second square bracket indicates the corresponding Fps of each tracker.

In this subsection, to give further comparisons, we test the proposed tracker on the recent benchmark [1]. This benchmark contains 25 recent state-of-the-art trackers, 51 different video sequences and two evaluation criteria, *i.e*., the precision plot and the success plot of the one-pass evaluation (OPE). Here, the precision plot shows the ratio of frames whose center location error (CLE) is within the given threshold. Additionally, the success plot indicates the percentage of frames whose overlap rate is larger than the given threshold. In addition, the precision score and the success score are applied for measuring the overall tracking performance. Specifically, the precision score is given by the score on a selected representative threshold (e.g., 20 pixels), and the success score is evaluated by the area under curve (AUC) of each tracker. The tracking performances (including Fps) of our method and other compared trackers are illustrated in Figure 6. For clarity, we only demonstrate the comparisons with 10 state-of-the-art tracking methods of the benchmark. Here, for all of the trackers, the precision (or success) scores are presented in the first square brackets, and the Fps data are shown in the second square brackets.

From Figure 6, it can be seen that our proposed tracker achieves satisfying tracking performances in terms of accuracy (*i.e*., Rank 2 in these evaluations). It should be noted that our tracker (Fps = 7) is more efficient than the SCM tracker (Fps = 0.5), and our tracking results on the MATLAB platform are comparable to the Struck tracker, whose implementation depends on the C/C++ platform.

## 6. Conclusions

In this paper, a novel tracking method based on ELM and sparse representation has been proposed. First, the ELM technique is applied for quickly distinguishing the target and background observations. With the trained ELM classification function, most of the candidate samples relevant to the backgrounds are discarded, which alleviates the calculation cost of the following sparse representation. Then, a new constrained sparse tracking model has been established, in which the ELM learning results are embedded as a manifold learning constraint term, which can further develop the combination of ELM and sparse representation and lead to better tracking results. Moreover, the APG method is utilized for getting the optimal solution (in matrix form) of the constrained sparse tracking model within several iterations. Additionally, the matrix form formulation allows the candidate samples to be calculated in parallel, thereby leading to its being superior in terms of tracking efficiency. Finally, experimental evaluations on challenging sequences demonstrate the efficiency and robustness of the proposed tracker.
